# Deaddicta® for maintenance treatment of Opioid-dependence: A six-month follow-up 

**DOI:** 10.22088/cjim.15.2.318

**Published:** 2024

**Authors:** Abdolali Moosavyzadeh, Farzaneh Ghaffari, Mohammad Bagher Saberizafarghandi, Majid Talafi Noghani, Hossein Hassanpour, Fatemeh Emadi, Fatemeh Alijaniha, Zahra Bahaeddin, Leila Nasiri, Razieh Jafari Hajati, Mohsen Naseri

**Affiliations:** 1 Department of Persian Medicine, School of Medicine, Isfahan University of Medical Science, Isfahan, Iran; 2 Traditional Medicine Clinical Trial Research Center, Shahed University, Tehran, Iran; 3 School of Traditional Medicine, Shahid Beheshti University of Medical Sciences, Tehran, Iran; 4 Department of Addiction, School of Behavioral Sciences and Mental Health, Tehran Institute of Psychiatry, Iran University of Medical Sciences, Tehran, Iran; 5 Department of Traditional Medicine, School of Persian Medicine, Shahed University, Tehran, Iran; 6 Department of Basic Sciences, Faculty of Veterinary Medicine, Shahrekord University, Shahrekord, Iran; 7 Department of Health Equity, Immunoregulation Research Center, Shahed University, Tehran, Iran; 8 Hikmat, Islamic and Traditional Medicine Department, The Academy of Medical Sciences, Tehran, Iran

**Keywords:** Craving, *Datura Stramonium* L, Opium dependence, Persian Medicine.

## Abstract

**Background:**

Opioid dependence, is one of the world's most critical health problems. Deaddicta is a herbal product considered an effective treatment for opioid addiction. Deaddicta's efficacy in the maintenance treatment of patients with opioid use disorder has recently been demonstrated through a double-blind randomized controlled trial (RCT). This study aimed to evaluate the permanence of Deaddicta's efficacy six months after the end of the maintenance treatment for opioid dependence.

**Methods:**

This study was performed following the previous RCT on the maintenance treatment of opioid addicts. Out of 41 participants who completed the study for three months in the previous research, 15 from the intervention group (Deaddicta capsules, 1500 mg/day) returned for follow-up. They all previously fulfilled the DSM-IV criteria for addiction, were aged 18 to 65, and had discontinued Deaddicta for six months. The outcome measures included addiction severity, depression and anxiety levels, and craving score. The scores of each parameter were compared in three phases: before intervention; after three months of intervention; and six months after the end of the study.

**Results:**

Depression, anxiety, and craving scores decreased six months after the end of the previous study. This decrease was significant in the craving score (*P* = 0.011). No significant increase was observed in the frequency of use. The regression analysis showed a negative relationship between craving and the progression of phases.

**Conclusion:**

The Deaddicta product may have desirable and effective properties in decreasing temptation and, as a result, the maintenance treatment of opioid dependence.

## Introduction

Drug abuse and opioid dependence are major health problems around the world. There are various therapeutic interventions for substance use disorders (1). Since patients with substance use disorders undergoing conventional pharmacological therapies may still experience relapse, adding complementary treatments such as medicinal plants may have promising effects in the treatment of addiction (2).

During recent decades, Iran has faced an increase in drug abuse and related consequences (3). In this regard, opium and its derivatives are among the most highly abused drugs that affect adults (3). The major problem in opioid use disorder treatment is craving and relapse (4). Agonist maintenance therapy (such as methadone) is a comprehensive treatment plan in which some medications are prescribed as a substitute for long-term opioid-dependent patients. This method has clinical benefits that should be considered along with its potential risks (5). However, withdrawal syndrome, craving, and cognitive deficits are some of its side effects that can be reduced with the help of natural and herbal compounds (6). 

The utilization of herbal compounds and complementary therapies for the treatment of disease and addiction is rapidly expanding. A trans-disciplinary endeavor called reverse pharmacology has recently emerged, and this new academic discipline can reduce three major bottlenecks—cost, time, and toxicity—frequently encountered in conventional drug development. Thus, medicine discovery based on reverse pharmacology follows a path from clinics to laboratories, the opposite direction applied for conventional synthetic drug development (7).

Traditional Persian medicine (TPM), a valuable resource of valid applied studies by ancient Iranian scholars, recommends numerous medicinal plants based on each patient’s characteristics and practices multiple target therapies (8-12). The Book of Afyuniyah is the most important Persian medical treatise on opium addiction and its treatment. TPM scientists knew the therapeutic effects and side effects of opium and opiates (13). Although in the documents of TPM, opium is used as an anti-pain, anti-diarrhea, and anti-cough medicine. It is called hypnotic and narcotic, but the addictive effects of this substance have been carefully considered and this disorder, sign and symptoms, side effects and its management are described in detail (14, 15). One of the herbal products that have been used in TPM to treat people addicted to opiates is the Hab-o Shefa product. This product is made of four herbs, including *Datura stramonium *L. seeds (43.3%), *Rheum palmatum* L. root (27.9%), *Zingiber officinale* Roscoe rhizome (14.4%), and *Acacia Senegal *L*.* gum (14.4%), which has been cited as an opioid withdrawal drug in TPM sources (11). Alkaloids, especially scopolamine and hyoscyamine, flavonoids, saponins, and phenols are the main chemical compounds in Hab-o Shefa (11). Several studies have been conducted on this product (8, 10, 11, 16, 17, 18).

The Hab-o Shefa product has been registered under the brand name Deaddicta^®^ with Iran Registration Code (IRC) 6640275830797081 in the Iran Food and Drug Organization. The effectiveness of Deaddicta in controlling withdrawal symptoms such as muscle aches, colic pain, rapid sedation, reducing anxiety and anger, and relieving depressive symptoms has been evaluated in previous studies. This compound was first introduced in the Kholasaye-e-Al-Tajarob book of the Great Tenth Century Author; According to Baha-Al-Dowlah Razi, this compound originated from his father's inventions (17). Since then, other scientists have used the remedy and reported many properties leading to the strengthening of the sensual ability and a departure from the abuse of opiates (18). Also, it has been mentioned in the opiate treatise of Emad al-Din Mahmoud Shirazi of Hab-o Shefa (17). Table 1 summarizes the pharmacological effects and mechanisms of action of Deaddicta compounds.

Toxicological data for Deaddicta showed no significant complications during its use up to 5000 mg/kg for 14 days (18). Acute toxicity has also been evaluated for this product (19). According to Nazari et al. (2013), Deaddicta significantly had better control over symptoms of withdrawal and depression than the placebo and clonidine groups and no side effects were observed (16). Considering the significant effect of Deaddicta on the maintenance treatment of opioid-dependent people and a significant reduction in relapse, craving, anxiety, and depression (11), this study investigated the permanence of Deaddicta's efficacy in the maintenance treatment of opioid dependence six months after treatment ended. 

**Table 1:** Pharmacological effects and action mechanism of main Deaddicta compounds

**Scientific name**	**Pharmacological effects**	**Mechanism of action**
***Datura stramonium L*** **.**	Anti-diarrheal (43), anti-inflammatory (44), antioxidant (45), analgesic (44), neurological activities healing (46), anticholinergic effect (47).	GABAA receptors (43), serotonergic system (48), elevation of BDNF expression and improvement of neuro-system function (49), modulatory effects monoaminergic and cholinergic neurotransmission systems (50), modulates activities of purinergic enzymes (51).
***Rheum palmatum L.***	Anti-inflammatory (52), detoxification effects, antioxidant, protective cerebral cortex neurons (53), analgesic, antidiarrheal (54).	Potential MAO inhibitory activity (55), anti-inflammatory activity via the PI3K-Akt-mediated NF-κB pathway. Upregulates the expression levels of DA, 5-HT, and NE by activating MEK/ERK signal pathway (56). Up-regulates GR and BDNF levels in the hippocampus (57).
***Zingiber officinale Roscoe***	Strong anti-oxidant, analgesic, anti-inflammatory, inhibitory action of ginger on prostaglandins, immuno-modulatory, modulate some biochemical pathways activated in chronic inflammation (39).	Inhibits the expression of TNF-α, IL-6, and IL-β (58). Increases the concentrations of 5-HT and DA; improves the expressions of the BDNF, NGF, TrkB, and TrkA in the hippocampus (59) induced neuropeptide Y (NPY) expression (60)

## Methods

**Study design, participants, and interventions:** This research is a follow-up study to a previous randomized controlled trial on the maintenance therapy of opioid dependence (11). According to the previous study, 41 patients were treated with Deaddicta^®^ (Sanabel Darue Co., Tehran, Iran) or placebo 500 mg capsules (TDS) for 12 weeks after detoxification during a double-blind clinical trial. In the current study, all of those patients were invited for evaluation six months after the end of the previous study. 

The same inclusion and exclusion criteria were applied as in the earlier study (11).The inclusion criteria for this investigation were as follows: Diagnostic and Statistical Manual of Mental Disorders-IV (DSM-IV) criteria for addiction include age between 18 and 65, willingness to participate in the study, good health, and informed consent. The following exclusion criteria were considered: alcoholism, a history of other psychiatric disorders, the use of psychiatric drugs such as sodium valproate and lithium, pregnancy, and breastfeeding, serious medical conditions such as glaucoma, urinary retention, epilepsy, Parkinson's, brain disease, heart and renal disease, allergic reactions to medical herbs, and the incidence of side effects.

**Clinical evaluation:** All study volunteers were checked out for vital signs, including blood pressure, pulse, and respiration rate, in three phases: before the intervention, after three months of intervention, and six months after the end of the study. To evaluate the patient's situation, the questionnaire forms were completed as follows:

**Addiction Severity Index, Lite Version (ASI-Lite):** The Addiction Severity Index, Lite version (ASI-Lite) is a shortened version of the Addiction Severity Index (ASI). The ASI-Lite questionnaire was used to assess the number of days of drug abuse (self-reported) in the past month (20). 

**Hamilton depression questionnaire:** The Hamilton Rating Scale for Depression (HRSD) is a multiple-item questionnaire designed for adults and is used to rate the severity of their depression by probing mood, feelings of guilt, suicide ideation, insomnia, agitation, anxiety, weight loss, and somatic symptoms. In this study, a form of 24 questions was used in which the score of each individual was in the range of 0 to 77. The reliability of this test through retesting was 0.85 and 0.89 (21).

**Hamilton anxiety questionnaire:** The Hamilton anxiety questionnaire (Hamilton Anxiety Rating Scale, HAM-A) is a psychological questionnaire used by clinicians to rate the severity of a patient's anxiety. The scale consists of 14 items with a score range of 0 to 56 and is designed to assess the severity of a patient's anxiety. The reliability of this test through retesting was 0.81 (22).

**Craving Believe Questionnaire (CBQ):** The CBQ is a self-report questionnaire, where patients rate their agreement with every 20 items on a 7-point Likert scale. This questionnaire measures beliefs about substance cravings. Higher scores indicate feeling more helpless to deal with cravings. To evaluate the reliability of this questionnaire, the internal homogeneity coefficient in terms of Cronbach's alpha (0.84) and the split-half method (0.81) were calculated (23). 

**Visual analog scale (VAS): **A VAS is a measurement instrument for subjective characteristics or attitudes that cannot be directly measured. When responding to a VAS item, respondents specify their level of agreement with a statement by indicating a position along a continuous line between two endpoints. According to the analysis, the scores of each parameter were compared in three phases: phase 1, before intervention; phase 2, after three months of intervention (Deaddicta), and phase 3, six months after the end of the study.

**Statistical analysis:** The data were represented as the mean ± standard deviation (SD). The significance level of *p* < 0.05 was considered in the analysis. A normality test (Kolmogorov–Smirnov test) was performed to determine the normal distribution of sample data in the population. To compare the mean of the variable with the normal distribution in the two groups, the independent t-test and, otherwise, the Mann-Whitney test were used. To compare the means in each group before and after the intervention, a paired t-test or Wilcoxon test was used. The relationship between the CBQ, VAS, depression, and anxiety questionnaires for five time periods was shown as regression curves. All statistical calculations were done with the computer program SPSS 26.0 software for Windows (IBM-SPSS, Inc., Chicago, IL, USA). The ethical code for this study is IR.SHAHED.REC.1397.003.

## Results

**Figure 1:**
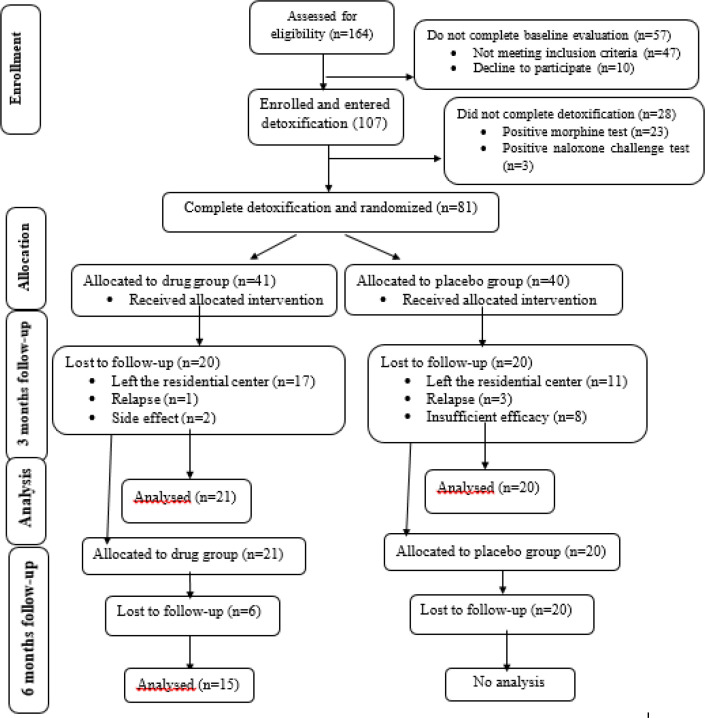
Characteristics of the participants, enrollment, allocation, and follow-up

Figure 1 is the participant flow diagram of the study on opioid dependence maintenance therapy (11). In the previous study, 80 patients were randomly divided into two groups and received Deaddicta or a placebo for three months.

 Forty-one patients completed the study and were included in the final analysis. After six months, we were able to find 15 participants in the intervention group. Therefore, analysis was only conducted on data from these 15 patients. Since no participants from the control group were available for follow-up, no inter-group comparisons were performed. 

Table 2 shows the demographic characteristics of the participants. Table 3 shows the mean and standard deviation for the ASI-Lite score in the three phases described above. The number of self-reported days of opioid use in the first and second phases was zero; however, a slight non-significant increase was observed six months after the end of the study (*P*= 0.118).

Table 4 shows the mean and standard deviation of scores for depression, anxiety, the CBQ, and the VAS questionnaires in the three phases. Table 5 compares changes in scores over the three time steps.

 The results indicated that the depression score in the treated patients after starting the intervention had a decreasing trend. The depression scores for each stage before the intervention, after the intervention, and six months after the end of the intervention were 9.2 ± 7.24-6.0 ± 4.08, and 4.5 ± 6.33, respectively. However, this reduction rate was not obvious. On the other hand, the depression score showed no significant change six months after the study ended (*P* = 0.081).

 In the case of anxiety, the score for each stage-before the intervention, after the intervention, and six months after the end of the intervention—was 12.7 ± 10.29, 12.2 ± 4.03. And 8.2 ± 6.69, respectively. The anxiety score in the third month after receiving the intervention did not differ from the beginning; however, in the six months following the end of the study, there was a significant decrease in the anxiety score (*P*= 0.038). 

**Figure 2:**
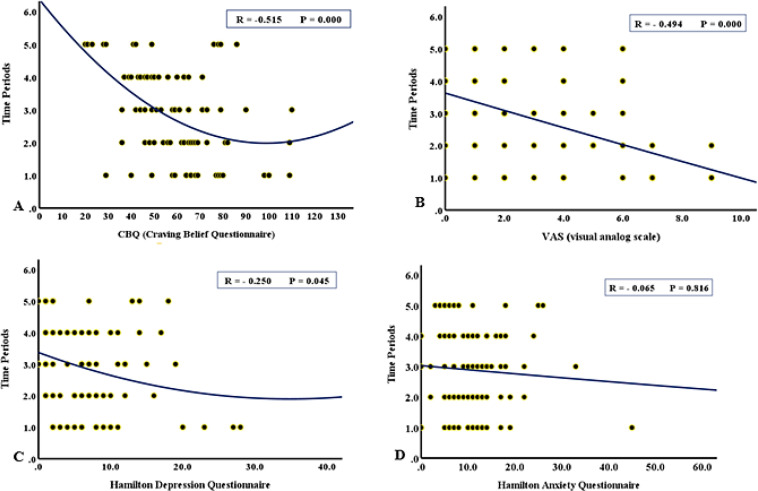
Evaluation (regression curve) of CBQ (A), VAS (B), depression (C), and anxiety (D) in 5 time periods: of the onset of consumption (1), the first month after consumption of Deaddicta (2), the second month after consumption of Deaddicta (3), the third month after consumption of Deaddicta (4) and 6 months after discontinuing Deaddicta consumption (5)

The craving score was assessed using the CBQ questionnaire, which decreased significantly three months after allocation to the intervention (*P*= 0.003) and also six months (*P*= 0.005) after the end of the study. The VAS questionnaire scores, which had a decreasing trend in the earlier study, demonstrated a slight increase six months after the study ended; however, this increase was not significant (*P*= 0.289). In addition, the relationship between CBQ, VAS, depression, and anxiety scores at five time periods is shown in figure 2 as regression curves. 

**Table 2:** Baseline demographic and clinical characteristics of participants

	**Variables**
32.5 (6.14)	**Age, mean (SD)**
9 (60)	**Married, n (%)**
1 (6.6)	**Unemployed, n (%)**
12 (80)	**High-school education or less, n (%)**
10.00 (4.24)	**Years of opioid use, mean (SD)**
6 (29.32)	**Lifetime IDU, n (%)**

**Table 3:** Average rates of drug abuse in different phases

**Mean ± SD**			
**Six months after the end of the study**	**After intervention**	**before intervention**	**Variable**
1.46 ± 3.12	0.00 ± 0.00	0.00 ± 0.00	**Days of opioid use**

**Table 4:** Mean score of depression, anxiety, and craving in the three phases in patients treated with Deaddicta

**Mean ± SD**			
**six months after the end of the study**	**After three months of intervention**	**before intervention**	**Variable**
4.5 ± 6.33	6.0 ± 4.08	9.2 ± 7.24	**Depression**
8.2 ± 6.69	12.2 ± 4.03	12.7 ± 10.29	**Anxiety**
39.1 ± 24.72	51.2 ± 10.23	74.7 ± 20.72	**Craving (CBQ)**
1.8 ± 2.23	1.2 ± 1.14	4.7 ± 3.06	**Craving (VAS)**

**Table 5:** Comparison of craving, depression, and anxiety in different phases

***P-value***				**Comparison step**	**Base stage**
**Craving (VAS)**	**Craving (CBQ)**	**Anxiety**	**Depression**		
0.001	0.003	0.864	0.246	**After three months of intervention**	**before intervention**
0.011	0.005	0.222	0.081	**six months after the end of the study**	
0.289	0.110	0.038	0.57	**six months after the end of the study**	**After three months of intervention**

## Discussion

Various herbal medicines have been mentioned in TPM for the treatment of opioid use disorder. One of these products is Deaddicta (Hab-o-shefa), which consists of several herbs. The results of the present follow-up study showed that the reducing effect of Deaddicta on cravings was maintained six months after the end of the previous study. Although anxiety and depression also decreased, they were not statistically significant. 

Deaddicta has been shown in various studies to reduce drug withdrawal syndrome and affect the main parameters of the maintenance treatment phase (8, 10, 11, 16, 17). In an animal study and clinical trial by Nazari et al. (2013), the efficacy of this drug in controlling morphine withdrawal symptoms was evaluated. 

They concluded that the control of withdrawal symptoms and depression in the Deaddicta group was better than in the other groups (8, 16). Our previous study indicated that treatment with Deaddicta for three months in twenty-one patients after detoxification improved their cravings, depression, and anxiety (11). 

Rahimi-Movaghar et al. (2011) followed-up on 79 patients six months after compulsory methadone maintenance treatment. Almost 23.5% of patients remained under treatment, and only 11.8% had a negative morphine test (24). In another study, 436 opioid addicts were treated and evaluated after six months of discontinuation, 64% of whom were in good health (25).

 In the study by Onofrio et al. (2017), six months after treatment discontinuation, the buprenorphine group did not differ significantly from the control group in drug use days or morphine urine tests (26). However, in some studies, follow-up results after treatment discontinuation indicated drug effectiveness stability. For example, in a study of Ibogaine in 2017, 8 of the 12 people included in the study were re-evaluated after 12 months for light-weight addiction index (ASI-lite) and depression. The results showed that the drug's effectiveness on these two parameters continued (27). Depression and anxiety are important concerns complicating addiction treatment. Individuals undergoing treatment for illicit drug use with anxiety and/or depression are more likely to continue substance use and suffer addiction consequences (28). A previous study reported that 57.5% of participants in methadone maintenance treatment suffer from depressive symptoms, and about 25.8% of them have suicidal ideation (29). 

Peles et al. (2007) also found that 50% of patients under treatment with methadone experienced depression. However, in this regard, Deaddicta could be a suitable replacement for methadone because of its helpful effect on the improvement of depression and anxiety (11, 30). One of the most important effects of Deaddicta is its ability to reduce cravings. The results of the current study show the persistence of craving reduction. Fareed et al. (2011) reviewed that despite the effectiveness and safety of methadone maintenance treatment for heroin dependence improvement, there are controversies about its effect on craving. Most studies reported that methadone has neutral or incremental effects on heroin craving, and patients on methadone maintenance treatment may still be at risk of cue-induced cravings (31).

It has been reported that *D. stramoniom* has anticholinergic, anesthetic, analgesic, sedative-hypnotic, anti-parkinsonian, and aphrodisiac qualities through its tropane alkaloids. Tropane alkaloids have various degrees of affinity for monoaminergic transporters and then exhibit different CNS effects related to the function of monoaminergic neurotransmitters (32).

In a study in 2022, it was reported that *D. stramonium* possesses anxiolytic- and antidepressant-like activities that could be due to higher monoaminergic turnover (serotonin and adrenaline) (33). Perviz et al. also indicated that some plant alkaloids could have antidepressant activity by inhibiting monoamine oxidase, increasing monoaminergic turnover, and reducing corticosteroids (34). 

In many studies, it has been confirmed that the anxiolytic and antidepressant activities of some herbal remedies (as reported for *D. stramonium*) may be related to their components such as flavonoids, saponins, alkaloids, tannins, and terpenoids (35). 

*Z* *.* *Officinale* is one of the other ingredients in Deaddicta, and some studies have proven its anxiolytic effects (36). Fadaki et al. in 2017 indicated that *Z. Officinale* extract can reduce anxiety reactions in a dose-dependent manner, and 200 mg/kg dose increased the movement activity compared to diazepam significantly. In-vitro and in-vivo animal models show ginger's anti-inflammatory activity (37). Recent studies' results have demonstrated a relationship between reducing relapse and anti-inflammatory molecules whose effects reach the brain. (38). Ginger's bioactive constituents inhibit the inflammation process by inhibiting arachidonic acid metabolism, cyclooxygenase, and lipoxygenase. They also inhibit leukotriene synthesis (39). 

Deaddicta's effect on craving reduction has not been studied before. The primary mechanism of efficacy of this product is unclear and needs further investigation. Despite current medications for addiction treatment, which mostly act through agonistic or ‎antagonistic mechanisms, Deaddicta probably has effects on opioid receptors regarding its antinociceptive (40) and hypnotic effects (41) and also acts through other possible mechanisms, ‎such as anticholinergic effects (42). 

The main limitation of the present study was that no patients from the control group were available for follow-up six months after the previous study. Therefore, despite the design of the previous study in two separate groups, only intra-group comparisons were performed in the current study. In this ‎study, it was observed that the effect of Deaddicta on craving reduction may remain to some ‎‎extent after the treatment is discontinued. Further studies are needed to investigate the molecular pathways of this effect.‎
